# Tumors Metastatic to Thyroid Neoplasms: A Case Report and Review of the Literature

**DOI:** 10.4061/2011/238693

**Published:** 2011-03-31

**Authors:** Todd M. Stevens, Alan T. Richards, Chhanda Bewtra, Poonam Sharma

**Affiliations:** ^1^Department of Pathology, Creighton University Medical Center, Omaha, NE 68131, USA; ^2^Division of Head and Neck Surgery, University of Nebraska Medical Center and Creighton University Medical Center, 42nd and Emile Street, Omaha, NE 68198, USA

## Abstract

Metastasis into a thyroid neoplasm—tumor-to-tumor metastasis—is exceedingly rare. We describe the 28th documented case of a tumor metastatic to a thyroid neoplasm and review the literature on tumor-to-tumor metastasis involving a thyroid neoplasm as recipient. All cases showed a recipient thyroid neoplasm with an abrupt transition to a morphologically distinct neoplasm. Metastasis into primary thyroid neoplasm was synchronous in 33% of cases and metachronous in 67%. Follicular adenoma was the most common recipient thyroid neoplasm overall (16/28), and papillary thyroid carcinoma was the most common malignant recipient neoplasm (9/28). Of the 9 recipient papillary carcinomas, 6 were follicular variants. Renal cell carcinoma was the most common neoplasm to metastasize to a primary thyroid neoplasm (9/28), followed by lung (6/28), breast (5/28), and colon (3/28) carcinoma. Tumor-to-tumor metastasis should be considered whenever a dimorphic pattern is encountered in a thyroid tumor.

## 1. Introduction

Metastasis to thyroid gland is uncommon, with reported incidences ranging from 0.5% [1] in unselected autopsy studies to as high as 24% [2, 3] in those with widespread metastatic disease. Malignant melanoma and carcinomas of lung, breast, kidney, gastrointestinal tract, and head and neck, among many other tumors, have been noted to secondarily involve the thyroid gland [1–4]. Metastasis to a primary thyroid neoplasm is extremely rare, however. To our knowledge, only 27 cases of tumor-to-tumor metastasis in which the recipient tumor was a primary thyroid neoplasm have been reported in the literature [3–23]. We report a case of poorly differentiated carcinoma of lung metastatic to a follicular adenoma and review the literature on tumor-to-tumor metastasis in the thyroid gland.

## 2. Case Report

**Figure 1:**
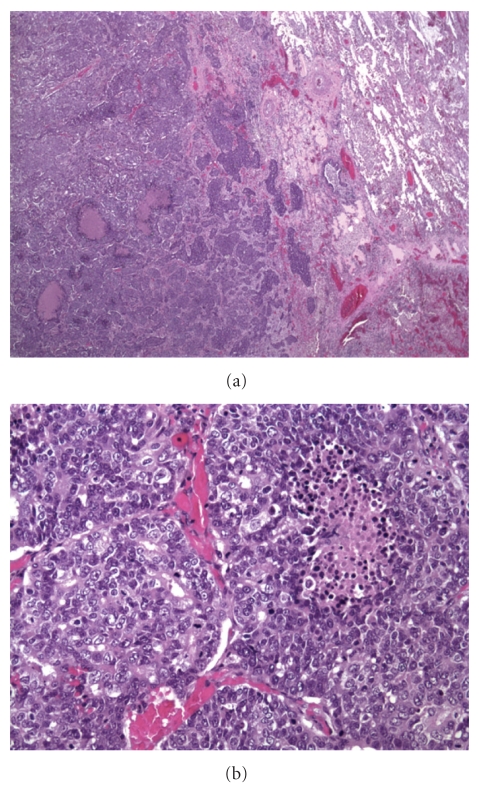
(a) Low power (20x; H & E) photomicrograph demonstrating invasive lung tumor arranged in nests and islands with abundant central necrosis. Cytologically (b) the malignant cells displayed enlarged, hyperchromatic nuclei with vesicular to coarse chromatin and a moderate amount of amphophilic cytoplasm (200x; H & E).

A 65-year- old white male with coronary artery disease presented to medical attention with shortness of breath. Imaging studies revealed a mass in the right upper lobe of lung, and a right upper lobectomy was performed. A peripherally located 5.5 × 5.0 × 4.5 cm irregular tan-white tumor causing pleural retraction was noted. Histologic exam revealed an invasive tumor arranged predominantly in nests and islands with abundant central necrosis (Figure 1(a)). A microscopic focus of glandular pattern was identified, but no definite squamous differentiation was seen. Cytologically the neoplastic cells had a moderate amount of amphophilic cytoplasm and enlarged hyperchromatic nuclei with vesicular to coarse chromatin and inconspicuous nucleoli (Figure 1(b)). Greater than 5 mitotic figures per high power field were noted. The tumor did not invade the visceral pleura. Several immunoperoxidase stains were performed using a standard streptavidin-biotin-peroxidase method to elucidate the tumor differentiation. The carcinoma cells showed focal positivity for cytokeratin 7 and cytokeratin 34 beta E12, but were negative for cytokeratin 5/6, p63, TTF-1, chromogranin, synaptophysin, and CD56 immunostains. Mucicarmine stain revealed no evidence of mucin production. A diagnosis of poorly differentiated carcinoma of lung with basaloid pattern was made. One regional lymph node and inferior pulmonary ligament tissue were involved by metastatic carcinoma. 

**Figure 2:**
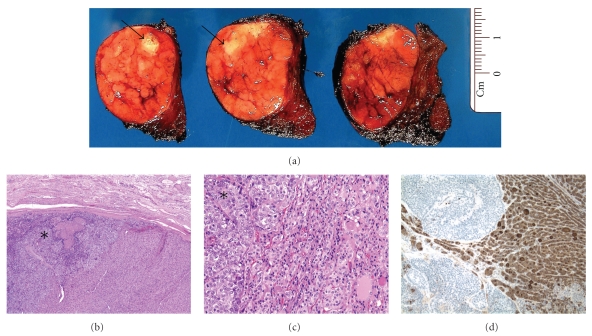
(a) An encapsulated tan-brown adenoma was present within the right thyroid lobe with multifocal white-tan areas within the adenoma (arrows). Within the encapsulated follicular adenoma is an abrupt transition to a morphologically distinct neoplasm (asterisk) (40x; H & E) (b). Metastatic carcinoma (asterisk) was arranged in nests with enlarged vesicular nuclei with inconspicuous nucleoli infiltrating the follicular adenoma (200x; H & E) (c). The metastatic carcinoma was negative for thyroglobulin, while the adenoma was strongly positive (100x; IHC) (d).

Postoperative tumor staging by positron emission tomography (PET) scan revealed an area of increased uptake in the right thyroid lobe (standardized uptake value: 64). Serum thyroid stimulating hormone (TSH), thyroxine, and calcitonin levels were not performed. Ultrasound guided fine needle aspiration of the thyroid lesion showed numerous microfollicles and scant colloid, consistent with a follicular neoplasm. No evidence of metastatic pulmonary poorly differentiated carcinoma was identified in the thyroid aspirate. A total thyroidectomy was performed approximately 8 weeks following the diagnosis of the primary lung carcinoma. Gross examination revealed a 36-gram thyroid gland with a 4.5 × 3.2 × 3.0 cm tan-brown encapsulated nodule in the right lobe. Within this nodule were multifocal irregular white-tan lesions (Figure 2(a)). Histologic exam of the nodule revealed a follicular adenoma with microfollicular and trabecular architecture; no capsular or vascular invasion was identified. Within the follicular adenoma were multifocal areas showing an abrupt transition to a morphologically distinct neoplasm comprised of cells with enlarged, hyperchromatic nuclei arranged in rounded nests with abundant central necrosis (Figure 2(b)). This tumor in the follicular adenoma was cytomorphologically identical to the patient's lung carcinoma (Figure 2(c)). Metastatic tumor emboli were also present in the intracapsular vessels of the follicular adenoma. Immunoperoxidase stains of these cells were negative for thyroglobulin (Figure 2(d)), calcitonin, and thyroid transcription factor (TTF-1). This metastatic carcinoma did not involve the nonneoplastic thyroid gland. Poorly differentiated carcinoma of lung metastatic to a follicular adenoma was diagnosed. There was no evidence of tumor in the left thyroid lobe. 

In addition, a 4.0 × 3.9 × 3.0 cm subcutaneous right chest wall mass excised at the time of thyroidectomy was completely replaced by metastatic poorly differentiated carcinoma of lung.

## 3. Discussion

Criteria used in diagnosing tumor-to-tumor metastasis require that the recipient tumor is a true neoplasm and that the donor neoplasm is a true metastasis, that is, invasion into the substance of recipient neoplasm is proven, with the caveat that the presence of only tumor emboli within a recipient neoplasm does not qualify as a true tumor-to-tumor metastasis [3, 19, 24]. Collision tumor, contiguous growth of one neoplasm into another adjacent neoplasm, and metastasis to a lymph node already involved by lymphoreticular malignancy are also excluded [19, 24]. Our case of poorly differentiated carcinoma of lung metastatic to a follicular adenoma meets these criteria of a true tumor-to-tumor metastasis. Defined in this way, approximately 150 case reports of tumor-to-tumor metastasis exist in the literature [22, 25]. Renal cell carcinoma, meningioma, and thyroid neoplasms are frequent recipients in tumor-to-tumor metastasis [22, 25, 26]. Common donor tumors in tumor-to-tumor metastasis include carcinomas of lung, breast, stomach, prostate, and thyroid, among others [25]. 

This is the 28th case reported in which a thyroid neoplasm served as the recipient tumor in a tumor-to-tumor metastasis. In all cases an abrupt transition to a morphologically distinct neoplasm was noted. Of these 28 cases, the recipient thyroid neoplasm was benign in 16 cases (follicular adenoma) ([4–8, 10–12, 15, 17–19, 23],and the present case) and malignant in 12 cases [3, 4, 9, 13, 14, 16, 20–22]. Of the 12 malignant recipient thyroid neoplasms, there were 9 papillary carcinomas [3, 4, 9, 20–22] (6 of which were follicular variants of papillary carcinoma [9, 21, 22]) and 3 follicular carcinomas [13, 14, 16] (2 of Hurthle cell type [13, 14]). Renal cell carcinoma was the most common donor neoplasm metastasizing to a primary thyroid neoplasm (9 cases) [3–5, 9, 12, 13, 15, 17, 22] followed by lung carcinoma (6 cases, including the present case) [6, 8–10, 20], breast carcinoma (5 cases, including one infiltrating lobular carcinoma) [4, 7, 18, 21, 22], and colon carcinoma (3 cases) [10, 14, 19]. Isolated single cases of prostate adenocarcinoma [7], melanoma [16], pancreatic neuroendocrine carcinoma [9], Burkitt-like lymphoma [23], and a malignant Phyllodes tumor [11] have also been reported to metastasize to a primary thyroid neoplasm. In two cases the donor malignancy remained clinically occult until it metastasized to a primary thyroid neoplasm (colonic adenocarcinoma [19] and renal cell carcinoma [5], both metastatic to follicular adenoma). In two cases diagnosis of a tumor-to-tumor metastasis was established at the time of fine needle aspiration based on the dimorphic appearance of the sample, one a breast carcinoma metastatic to a follicular variant of papillary carcinoma [21] and the other a renal cell carcinoma metastatic to an oncocytic carcinoma [13]. Women outnumbered men in this series (16 women; 10 men; two cases did not specify sex), and the average age at presentation of tumor-to-tumor metastasis was 59.2 years (age range 38–82 years).

The interval between diagnosis of primary tumor and its metastasis into a thyroid neoplasm was noted in 24 cases ([3, 5–19, 22, 23], and the present case). Diagnosis of primary tumor and its metastasis into a thyroid neoplasm were synchronous in 8/24 (33%) cases and metachronous in 16/24 (67%) cases (interval range of 2 months–10 years). In 13 cases, presence or absence of metastasis into nonneoplastic thyroid gland could be ascertained from the case reports ([3, 6, 9, 10, 13, 15, 19, 22], and the present case). In 10 of these cases (10/13; 77%), metastasis was confined to the primary thyroid neoplasm. Presence or absence of metastasis to tissue other than the thyroid gland could be determined in 20 cases ([3, 6, 7, 9–11, 13, 14, 16–19, 22, 23], and the present case), seventeen of which (17/20; 85%) also showed metastasis of donor tumor to anatomic locations other than the thyroid gland. Outcome after diagnosis of tumor-to-tumor metastasis was available in 15 cases ([3, 5–7, 9–11, 17, 19, 22, 23], and the present case). Five of these (5/15) patients died at the time of, or shortly after, the tumor-to-tumor metastasis, and 1 patient (1/15) died 2 years after diagnosis of tumor-to-tumor metastasis. 9/15 patients were alive at the time of the case reports. 

Neoplastic thyroid gland may be fertile “soil” for tumor metastasis due to decreases in oxygen concentration, altered iodine content, and the rich vascularity associated with neoplasia [3]. Tumor metastasis is very complex, and tumor-to-tumor metastasis is likely even more so, and these theories are probably only part of a complex mechanism that at present is poorly understood. 

Tumor-to-tumor metastasis should be considered whenever a distinct, dimorphic pattern is encountered in a tumor [19, 25]. When a component of poorly differentiated carcinoma is present in the thyroid in association with a follicular patterned lesion, as occurred in this case, the differential diagnosis would include thyroid carcinoma arising in association with a follicular adenoma, insular carcinoma (poorly differentiated thyroid carcinoma), glandular variant of medullary carcinoma, the rare mixed medullary and follicular composite tumor, mucoepidermoid carcinoma, contiguous spread from carcinomas of adjacent pharynx, larynx, trachea, or esophagus, and metastasis [4, 7, 9, 27]. Colon carcinoma metastatic to the thyroid gland and columnar cell carcinoma of the thyroid must be distinguished from one another [28]. Metastatic carcinoma within the thyroid gland is negative for thyroglobulin and calcitonin [4, 7]. Thyroid transcription factor-1 (TTF-1) has little value in the differential diagnosis of lung carcinoma metastatic to thyroid versus a primary thyroid carcinoma, as both are often positive [20]. Similarly, both metastatic lung carcinoma and glandular variant of medullary carcinoma can be CEA positive. Metastatic breast carcinoma can be ER and PR positive, but will be thyroglobulin and calcitonin negative [7, 18]. As in the present case, careful attention to clinical history, the dimorphic histologic appearance, comparison with prior surgical material, and immunohistochemistry for thyroglobulin, TTF-1, and calcitonin can be helpful in arriving at a correct diagnosis. 

Before diagnosing renal cell carcinoma metastatic to the thyroid gland, primary thyroid lesions that can show clear cell change should be excluded [4]. These include primary thyroid tumors such as follicular neoplasms (Hurthle cell type and otherwise), papillary carcinoma, medullary carcinoma, and paraganglioma. Attention to clinical history, presence of multifocal growth pattern, optically clear rather than granular cytoplasm, a sinusoidal pattern of vascularization, and positivity for CD10, renal cell carcinoma antigen (RCC), and vimentin, and negativity for TTF-1 and thyroglobulin, favor a diagnosis of metastatic renal cell carcinoma [3, 5, 17]. Other secondary tumors with clear cell features should also be excluded, including those arising from the lung and salivary gland tissue. Distinction of a primary tumor from metastasis can be difficult without histologic comparison with prior specimen. Some have used DNA ploidy, loss of heterozygosity (LOH) analysis of the von Hippel-Lindau (VHL) gene locus, and Ras mutation status as adjuncts to diagnosing tumors metastatic to thyroid [7, 22]. Presence of Ras mutation favors a primary thyroid follicular neoplasm over metastatic renal cell carcinoma. Loss of VHL gene locus favors a diagnosis of metastatic renal cell carcinoma over a primary thyroid neoplasm with clear cell change [22]. 

This paper reviews the 27 reported cases of a thyroid neoplasm serving as recipient in a tumor-to-tumor metastasis and describes the 28th such case, a poorly differentiated carcinoma of lung metastatic to a follicular adenoma. It is important to note, however, that even in patients with a clinical history of malignancy a new thyroid nodule is still likely to be a benign lesion [17]. While metastatic tumors in the thyroid gland are relatively uncommon, tumors metastatic to other thyroid neoplasms are extremely rare. Clinical history of prior malignancy, presence of a dimorphic cell population, and comparison with prior surgical material is useful in diagnosing tumor-to-tumor metastasis involving the thyroid gland as recipient. This review of 28 examples of a thyroid neoplasm serving as recipient tumor in a tumor-to-tumor metastasis indicates that follicular adenoma is the most common recipient thyroid neoplasm (16/28 cases), and papillary carcinoma is the most common malignant recipient thyroid neoplasm (9/28 cases). Renal cell carcinoma, lung, and breast carcinomas are the most common neoplasms to metastasize to a thyroid neoplasm.
